# Tracking Design of an Uncertain Autonomous Underwater Vehicle with Input Saturations by Adaptive Regression Matrix-Based Fixed-Time Control

**DOI:** 10.3390/s22093385

**Published:** 2022-04-28

**Authors:** Hsiu-Ming Wu

**Affiliations:** Department of Intelligent Automation Engineering, National Taipei University of Technology, Taipei 10608, Taiwan; hmwu@mail.ntut.edu.tw

**Keywords:** underwater autonomous vehicle, trajectory-tracking control, auxiliary sliding surface, fixed-time stability

## Abstract

In this study, a simplified model of an autonomous underwater vehicle (AUV) with input saturation based on kinematic and dynamic equations was built. Subsequently, a simplified model of the AUV was used to represent its main dynamic features. In terms of trajectory tracking, only the system’s structure (i.e., the regression matrix, which is flexible and non-unique) from the nominal model of the transformed system was required to design the proposed adaptive regression matrix-based fixed-time controller (ARM-FTC). A nonlinear auxiliary sliding surface was contained in the control design to shape the system’s frequency response. When the operating point was in the neighborhood of the zero auxiliary sliding surface, nonlinear filtering gains were increased to accelerate its tracking ability. Furthermore, the skew-symmetric property condition of the time-derivative of the inertia matrix and the Coriolis and centrifugal force matrices was not necessitated for the controller design. Under an appropriate condition for lumped uncertainties, the fixed-time convergence of the auxiliary sliding surface and the corresponding tracking error is guaranteed to go to zero by the Lyapunov stability theory. Finally, a comparative study was conducted through simulations for the AUV with external disturbance and input saturation among the known parameters, learning parameters reflecting a regression matrix, and another asymptotical robust tracking control scheme. The results validate the fast tracking ability of a desired time-varying trajectory of the proposed control scheme.

## 1. Introduction

In recent years, more and more practical applications are conducted using AUV systems, e.g., reconnaissance, inspection of underwater pipelines [1], route exploration, search and rescue, etc. For an effective execution of the aforementioned missions, trajectory-tracking control of AUVs is a necessary function in these types of applications. Different control schemes are available in the literature to control AUVs including adaptive control [2,3,4], fuzzy control [5,6], model predictive control [7,8], and backstepping control [9,10], which are either completely model-based or model-free controllers. The proposed control scheme belongs to a semi-model-based control strategy whose structure is based on a regression matrix of the AUV model, and its system parameters are estimated via designed adaptive laws. Moreover, the presented control scheme does not require a priori knowledge of the uncertainty bounds. Control of AUVs has been the subject of a lot of research activities in the past decade, and a representative few are discussed here.

Karkoub et al. [11] presented a hierarchical robust nonlinear (HRN) control for the trajectory tracking of an AUV subject to uncertainties. The proposed HRN control scheme utilized the backstepping and sliding-mode techniques simultaneously with a hierarchical structure based on the kinematic and dynamic models of the system. Computer simulations have shown that the overall closed-loop system achieves good asymptotic tracking performance. In addition, a 2D and 3D integral line-of-sight (ILOS) guidance method for path-following tasks of under-actuated AUVs in the presence of constant irrotational ocean currents was proposed in [12]. Simulation and experimental results validated the effectiveness of the proposed method. Peng et al. [13] designed a path-following control scheme including guidance and control loops for an under-actuated AUV system subject to velocity and input constraints. To connect the guidance and control loops, a reference governor is formulated as a quadratically constrained optimization problem. Then, a projection neural network is used to attain the optimization in real time. Simulation results demonstrated the effectiveness of the proposed method. An adaptive second-order fast nonsingular terminal sliding mode control scheme was presented in [14] for the trajectory tracking of fully actuated AUVs in the presence of dynamic uncertainties and time-varying external disturbances. The control scheme does not require prior knowledge of the upper bound of the system uncertainties and eliminates the chattering without reducing the tracking precision. The simulation results demonstrated the superiority of the technique over existing second-order nonsingular terminal sliding mode manifolds. In recent years, artificial intelligence (AI)-based control techniques have increased in popularity due to their suitability to many industrial applications including AUVs. Multi pseudo Q-learning was proposed by Shi et al. [15], which utilized “sub-greedy” policy to replace the greedy policy in Q-learning for continuous action spaces. The proposed scheme can reduce the overestimation of the action value function and stabilize the learning process. In addition, the deterministic policy gradient method is used to update the learning weights. The results show high-level tracking control accuracy and stable learning when applied to an AUV system. In [16], a high-order sliding mode controller with a disturbance observer was proposed to resist the negative impact of both parametric and bounded external disturbances. The authors validated the effectiveness of the control scheme experimentally. In [17], the authors designed an adaptive neural observer to estimate the dynamic uncertainties such that these are compensated in both the kinematics and dynamics.

Based on the above surveys and discussions, determining a comprehensive precise AUV model is one of the main challenges for an AUV control system due to its complex dynamics and high nonlinearity. The proposed adaptive regression matrix-based fixed-time control (ARM-FTC) is derived using newly transformed dynamics through its kinematic and dynamic models, such that the exact and completely known AUV dynamics are not required. In contrast, only the regression matrix of a simplified nominal AUV control system is needed to design an effective and robust controller. Furthermore, the regression matrix is flexible and not unique. The proposed ARM-FTC is capable of controlling the AUV systems subject to input saturation and system uncertainties caused by disturbance and unmodeled dynamics resulting from model simplification. From the outset, a nonlinear auxiliary sliding surface is designed so that the fixed-time convergence of the auxiliary sliding surface and then zero tracking error is guaranteed. Moreover, the proposed adaptive law with projection features can guarantee the boundedness of the learning parameters reflecting a regression matrix.

In summary, the main contributions of the work presented here are as follows: (i) No a priori knowledge of the system model is required to design the ARM-FTC controller. Instead, a flexible and non-unique regression matrix for the system structure from a simplified transformed model, obtained by combining the kinematic and dynamic models, is needed [18,19]. (ii) An adaptive regression matrix-based fixed-time control can learn the parameters reflecting a regression matrix such that the system uncertainties can be tolerant to achieving satisfactory trajectory tracking. The performance of the ARM-FTC is compared to those of a control system with known parameters reflecting a regression matrix and another robust asymptotic tracking control technique. (iii) Under an appropriate condition for lumped uncertainties, the fixed-time convergence to auxiliary sliding surface and then tracking error is obtained by Lyapunov stability theory, which is different from the finite-time bounded control technique [20]. It is worth noting that no disturbance observer is required to deal with extraneous disturbances [16,17,21].

The remainder of the paper is organized as follows: In Section 2, the modeling of an AUV and the problem formulation are given. The adaptive regression matrix-based fixed-time control design and its stability proof are described in Section 3. The simulation results and discussions are presented in Section 4. Finally, the conclusions and future studies are given in Section 5.

## 2. System Modeling and Problem Formulation

### 2.1. System Modeling

A typical AUV system has six DOFs including three coordinate positions and three orientations in space. The dynamic model comprises hydrodynamic features as well as uncertainties, which make the controlled system highly nonlinear and complex. In this section, simplified kinematic and dynamic models of an AUV for representing nominal system characteristics are developed to transform the original control system for the proposed control design.

(1) *Kinematic model*

**Figure 1:**
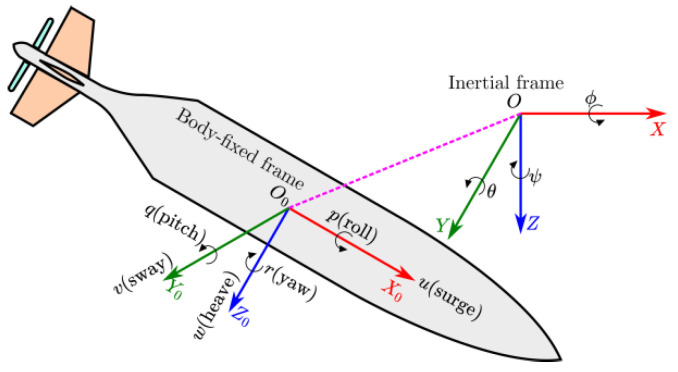
An AUV coordinate system.

The two coordinate frame systems of the AUV are illustrated in Figure 1: the inertial frame system {O−XYZ} and the body-fixed frame system $\left\{O_{0}-X_{0}Y_{0}Z_{0}\right\}$ in a three-dimensional Cartesian workspace. The kinematic subsystem of the AUV can be expressed as follows:

(1) $$\dot{\eta }=J(\eta )q$$

where $\eta ={\left[\begin{array}{cc} \eta_{1}^{T} & \eta_{2}^{T} \end{array}\right]}^{T};$ $\eta_{1}={\left[\begin{array}{ccc} x & y & z \end{array}\right]}^{T}$ and $\eta_{2}={\left[\begin{array}{ccc} \phi & \theta & \psi \end{array}\right]}^{T}$ represent the position and the orientation vector with respect to the inertial frame; $q={\left[\begin{array}{cccccc} u & v & w & p & q & r \end{array}\right]}^{T}$ is the translational and angular velocities vector with respect to the body-fixed frame; $J\in {ℜ}^{6\times 6}$ is the spatial transformation matrix between the inertial frame and the body-fixed frame.

(2) *Dynamic model*

The dynamic model of the AUV can be expressed in a compact vector form as [14]:

(2) $$M\dot{q}+C\left(q\right)q+D\left(q\right)q+g\left(\eta \right)+\tau_{u}=\tau$$

where $M\in {ℜ}^{6\times 6}$ is the inertia matrix including the rigid body matrix and the added mass matrix; $C\left(q\right)\in {ℜ}^{6\times 6}$ denotes the matrix of Coriolis and centrifugal forces including the rigid body matrix and the added mass matrix caused by the hydrodynamic effect; $D\left(q\right)\in {ℜ}^{6\times 6}$ is the hydrodynamic damping matrix including the linear and quadratic drag vectors; $g(\eta )\in {ℜ}^{6}$ represents the vector of gravity and buoyancy forces; $\tau \in {ℜ}^{6}$ is the control forces and moments vector, i.e., propulsion forces and moments acting on the center of mass of the AUV; $\tau_{u}\in {ℜ}^{6}$ is the uncertain current disturbance.

Since the simplified system of four degrees of freedom (i.e., p=q=0 and ϕ=θ=0) can capture dominant dynamics of the AUV system, how to design an effective and robust controller using the simplified model to deal with the original complex dynamic system becomes a challenging task. Under this design philology, the above matrices in (2) are simplified as $\eta ={\left[\begin{array}{cccc} x & y & z & \psi \end{array}\right]}^{T}\in {ℜ}^{4},$ $q={\left[\begin{array}{cccc} u & v & w & r \end{array}\right]}^{T}\in {ℜ}^{4},$ $J\in {ℜ}^{4\times 4},$ $M\in {ℜ}^{4\times 4},$ $C(q)\in {ℜ}^{4\times 4},$ $D(q)\in {ℜ}^{4\times 4},$ and $g(\eta )\in {ℜ}^{4}.$ Here,

$$J=\left[\begin{array}{cccc} \cos\psi & -\sin\psi & 0 & 0\\ \sin\psi & \cos\psi & 0 & 0\\ 0 & 0 & 1 & 0\\ 0 & 0 & 0 & 1 \end{array}\right],$$

$$M=\left[\begin{array}{cccc} m-X_{\dot{u}} & 0 & 0 & 0\\ 0 & m-Y_{\dot{v}} & 0 & 0\\ 0 & 0 & m-Z_{\dot{w}} & 0\\ 0 & 0 & 0 & I_{z}-N_{\dot{r}} \end{array}\right],\;C(q)=\left[\begin{array}{cccc} 0 & -mr & 0 & 0\\ -mr & 0 & 0 & 0\\ 0 & 0 & 0 & 0\\ mv & 0 & 0 & -mu \end{array}\right],\;$$

$$D(q)=\left[\begin{array}{cccc} -X_{u} & 0 & 0 & 0\\ 0 & -Y_{v} & 0 & 0\\ 0 & 0 & -Z_{w} & 0\\ 0 & 0 & 0 & -N_{r} \end{array}\right],\;\mathrm{and}$$

(3) $$g(\eta )={\left[\begin{array}{cccc} -X_{uu}u\left|u\right| & -Y_{vv}v\left|v\right| & -Z_{ww}w\left|w\right| & -N_{rr}r\left|r\right| \end{array}\right]}^{T}.$$

Since the simplified model has four DoFs with second-order behavior, the following eight states are defined as follows: $x_{1}=x,$ $x_{2}=y,$ $x_{3}=z,$ $x_{4}=\psi ,$ $x_{5}=u,$ $x_{6}=v,$ $x_{7}=w,$ and $x_{8}=r$. The symbols of the entries in the above-mentioned matrices are described in Table 1 [11]. Note that the off-diagonal terms of the drag matrices can be neglected because the hydrodynamic coupling is insignificant at low speeds. In addition, the buoyancy is typically trimmed so that it is approximately equal to the gravitational force [11,22].

(3) *Transformed system model*

In this subsection, the transformed model can be developed using the above kinematic and dynamic models. First, one can solve for q from kinematic model (1) as

(4) $$q=J^{-1}(\eta )\dot{\eta }$$

Its derivative with respect to time becomes

(5a) $$\dot{q}=J^{-1}(\eta )\ddot{\eta }+\frac{d}{dt}\left(J^{-1}(\eta )\right)\dot{\eta }$$

Knowing the fact $d(J^{-1})/dt=-J^{-1}\dot{J}J^{-1}$ and substituting it into (5a) yield

(5b) $$\dot{q}=J^{-1}\ddot{\eta }-J^{-1}\dot{J}J^{-1}\dot{\eta }$$

Replacing the expressions of q and $\dot{q}$ into the simplified dynamic model of the AUV (2), the transformed system model with the consideration of input saturation can be written as

(6) $$\overset{⌢}{M}\ddot{\eta }+F(q)\dot{\eta }+\overset{⌢}{g}(\eta )+{\overset{⌢}{\tau }}_{u}=sat(\overset{⌢}{\tau })$$

where $F(q)=-\overset{⌢}{M}\dot{J}J^{-1}+\overset{⌢}{C}(q)+\overset{⌢}{D}(q)\in {ℜ}^{4\times 4};$ $\overset{⌢}{M}=JMJ^{-1}$; $\overset{⌢}{C}=JCJ^{-1}$; $\overset{⌢}{D}=JDJ^{-1}$; $\overset{⌢}{g}=Jg\;$;$\overset{⌢}{\tau }=J\tau$; ${\overset{⌢}{\tau }}_{u}=J\tau_{u}$ and J is invertible; each component of $sat(\overset{⌢}{\tau })$ satisfies $sat({\overset{⌢}{\tau }}_{i})={\overset{⌢}{\tau }}_{i},i=1,2,3,4$, if $\left|{\overset{⌢}{\tau }}_{i}\right|\leq {\overset{⌢}{\tau }}_{s,i}$ where ${\overset{⌢}{\tau }}_{s,i}$ denotes a saturation value; $sat({\overset{⌢}{\tau }}_{i})={\overset{⌢}{\tau }}_{s,i}sign({\overset{⌢}{\tau }}_{i})$, otherwise. Moreover, ${\overset{⌢}{\tau }}_{u}$ is caused by the uncertain torque, and it is also represented as unmodeled dynamics in the simplified model (3). The transformed system obtained through kinematic model (1) and simplified dynamic model (3) is represented by (4), (5b), and (6).

### 2.2. Problem Formulation

The following nominal system structure of a simplified AUV model indicates that its DOFs and configuration must be realized such that an approximation by a suitable regression matrix multiplied by an unknown vector is achieved as follows:

(7) $$\overset{⌢}{M}\ddot{\eta }+F(q)\dot{\eta }+\overset{⌢}{g}(\eta )=Z(\eta ,\dot{\eta },\ddot{\eta })\bar{\phi }$$

where $Z(\eta ,\dot{\eta },\ddot{\eta })\in {ℜ}^{4\times r}$ is a known and non-unique regression matrix, and $\bar{\phi }\in {ℜ}^{r\times 1}$ is an unknown and bounded vector related to the moment of inertia, Coriolis and centrifugal forces, and hydrodynamic damping term as well as gravity and buoyancy forces. In fact, the number of unknowns $\bar{\phi },\;i.e.,\;r$ is flexible. It is the main feature of this study. Moreover, the transformed matrices $\overset{⌢}{M},$ F(q), and $\overset{⌢}{g}(\eta )$ have the following properties [18,19,23]:

P1: As $\dot{\overset{⌢}{M}}-2F(q)$ is a skew-symmetric matrix, i.e., $s^{T}(t)\left[\dot{\overset{⌢}{M}}-2F(q)\right]s(t)=0.$
P2: $\left\|F(q)\right\|\leq c_{0}\left\|\dot{\eta }\right\|$, where $c_{0}$ is a positive constant.
P3: $\left\|\overset{⌢}{g}(\eta )\right\|\leq c_{1}$, where $c_{1}$ is a positive constant.

Property P1 is not always satisfied. It can be modified and represented as:

P4: $\dot{\overset{⌢}{M}}-2\chi F(q)=X_{ss}(q)+X_{nss}(q),$ where $X_{ss}(q)$ and $X_{nss}(q)$ represent skew-symmetric and non-skew-symmetric matrices, respectively, and χ is a scalar chosen such that $X_{nss}(q)$ is minimized.
P5: This is obtained from property P2, $\left\|X_{nss}(q)/2+\chi F(q)\right\|\leq c_{2}\left\|\dot{\eta }\right\|,$ where $c_{2}$ is a positive constant.

**Figure 2:**
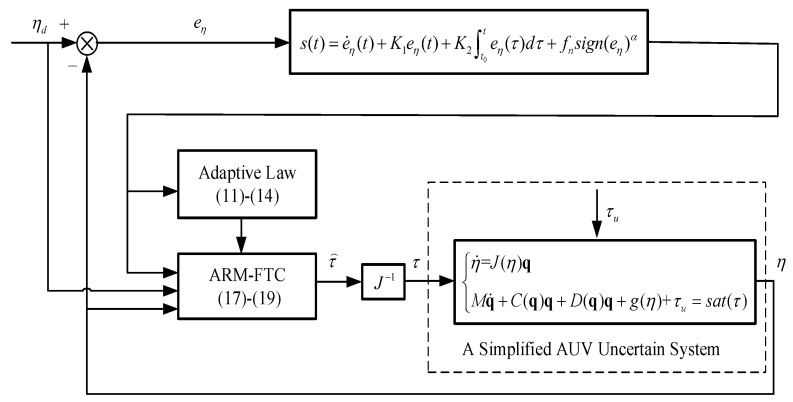
Overall control block diagram of a simplified AUV uncertain system.

The control problem is to design the ARM-FTC control $\overset{⌢}{\tau }$ for the transformed system models (4), (5b), and (6) in the presence of uncertain torque $\tau_{u}$. $\tau_{u}$ results from the simplification of model (3) are subject to saturated control input ${\overset{⌢}{\tau }}_{s}={\left[{\overset{⌢}{\tau }}_{s,i}\right]}_{i=1,2,3,4},$ so that the system output η tracks a desired trajectory $\eta_{d}$ in fixed time. Finally, the true control input τ is achieved using the following transformation: $\tau =J^{-1}\overset{⌢}{\tau }$. The closed-loop control diagram is depicted in Figure 2.

## 3. Adaptive Regression Matrix-Based Fixed-Time Controller Design and Stability Analysis

In this section, the details of the proposed ARM-FTC design are specifically described. Then, its stability analysis of the overall AUV control system is addressed via Lyapunov stability criteria.

### 3.1. Controller Design

Before designing the proposed ARM-FTC, a dynamic system with fixed-time stability is described in the following lemma [24].

**Lemma** **1** *If a smooth positive Lyapunov function V(t) satisfying $\dot{V}(t)+\lambda_{1}V(t)+\lambda_{2}V^{\gamma }(t)\leq 0$ for any $t\geq t_{0}$ where $\lambda_{1}\geq 0$, $\lambda_{2}>0$ and 0<γ<1, then the system will be stabilized in finite time and the setting time t of the system is given by*

(8) $$t\leq t_{0}+\ln\left[1+\lambda_{1}V^{1-\gamma }(t_{0})/\lambda_{2}\right]/\left[\lambda_{1}(1-\gamma )\right]\;$$

*where $V(t_{0})$ is the initial value of V(t).*

*Next, the proposed ARM-FTC is used to attain the fixed-time trajectory tracking of a simplified AUV uncertain system. A nonlinear auxiliary sliding surface is designed as follows:*

(9) $$s(t)={\dot{e}}_{\eta }(t)+K_{1}e_{\eta }(t)+K_{2}\int_{t_{0}}^{t}e_{\eta }(\tau )d\tau +f_{n}sign{\left(e_{\eta }\right)}^{\alpha }$$

*where $s(t)={\left[\begin{array}{cccc} s_{1} & s_{2} & s_{3} & s_{4} \end{array}\right]}^{T}\in {ℜ}^{4}$; $e_{\eta }(t)=\eta_{d}(t)-\eta (t)$ is a tracking error, and $\eta_{d}(t)$ is the desired trajectory; $K_{1}\in {ℜ}^{4\times 4}$ and $K_{2}\in {ℜ}^{4\times 4}$ are positive diagonal matrices, which are selected such that the auxiliary tracking error is stable; $f_{n}\in {ℜ}^{4\times 4}$ is a positive diagonal matrix to achieve the finite-time tracking performance; and 0≤α<1. Suppose that the nominal system structure satisfies the following result:*

(10) $$\overset{⌢}{M}\left(\ddot{\eta }+\dot{s}\right)+F(q)\left(\dot{\eta }+s\right)+\overset{⌢}{g}(\eta )=Z(\eta ,\dot{\eta },\ddot{\eta })\bar{\phi }$$

*where $Z(\eta ,\dot{\eta },\ddot{\eta })$ is a known regression matrix, and $\bar{\phi }$ denotes an unknown and bounded vector, which is obtained from the following online adaptive law:*

(11) $$\dot{\hat{\phi }}(t)=\left\{ \begin{array}{l} \phi_{a}(t)+\phi_{u}(t),\;\mathrm{if}\;\hat{\phi }(t)>{\bar{\phi }}_{M}\\ \phi_{a}(t),\;\;\;\;\mathrm{if}\;{\hat{\phi }}_{m}(t)\leq \hat{\phi }(t)\leq {\bar{\phi }}_{M}\\ \phi_{a}(t)+\phi_{l}(t),\;\mathrm{if}\;\hat{\phi }(t)<{\bar{\phi }}_{m}\; \end{array}\right.$$

*where*

(12) $$\phi_{a}(t)=\Psi \left[Z^{T}(\eta ,\dot{\eta },\ddot{\eta },s,\dot{s})s(t)-\hat{\phi }(t)\sigma^{T}s(t){\left\|s(t)\right\|}^{\beta }\right]$$

(13) $$\phi_{u}(t)=\delta \;\Psi {\left\|s(t)\right\|}^{1+\beta }\left(\hat{\phi }(t)-{\bar{\phi }}_{M}\right)/\left[\varepsilon +\left\|\hat{\phi }(t)-{\bar{\phi }}_{M}\right\|\right]\;$$

(14) $$\phi_{l}(t)=\delta \;\Psi {\left\|s(t)\right\|}^{1+\beta }\left(\hat{\phi }(t)-{\bar{\phi }}_{m}\right)/\left[\varepsilon +\left\|\hat{\phi }(t)-{\bar{\phi }}_{m}\right\|\right]\;$$

*Here, δ, ε >0, 0<β<1, ${\bar{\phi }}_{M}=\max\left\{\bar{\phi }\right\}$ and ${\bar{\phi }}_{m}=\min\left\{\bar{\phi }\right\}$ are assumed to be known, $\Psi \in {ℜ}^{r\times r}$ represents the adaptive gain, which is a positive diagonal matrix, and $\sigma^{T}\in {ℜ}^{1\times 4}$ denotes the e-modification gain whose entries are positive. The following lemma states a property used for the stability proof afterwards.*

**Lemma** **2.** *A property can be obtained as follows:*

(15) $$\begin{array}{ll} {\tilde{\phi }}^{T}(t)\hat{\phi }(t) & =\left[{\left\|\bar{\phi }\right\|}^{2}-{\left\|\tilde{\phi }(t)\right\|}^{2}-{\left\|\hat{\phi }(t)\right\|}^{2}\right]/2\\ & \leq \left[{\left\|\bar{\phi }\right\|}^{2}-{\left\|\hat{\phi }(t)\right\|}^{2}\right]/2\leq \left[{\left\|{\bar{\phi }}_{M}\right\|}^{2}-{\left\|\hat{\phi }(t)\right\|}^{2}\right]/2 \end{array}$$

*where $\tilde{\phi }(t)=\bar{\phi }-\hat{\phi }(t)$. Since adaptive law (11) can ensure ${\bar{\phi }}_{m}\leq \hat{\phi }(t)\leq {\bar{\phi }}_{M}$ for all t, there exists a positive constant ζ satisfying the following inequality:*

(16) $${\tilde{\phi }}^{T}(t)\hat{\phi }(t)\leq \zeta \;\forall t.$$

*At the final stage, the ARM-FTC is designed as follows:*

(17) $$\overset{⌢}{\tau }(t)=Z(\eta ,\dot{\eta },\ddot{\eta },s,\dot{s})\hat{\phi }(t)+\Gamma_{1}\left(\left\|s\right\|\right)s(t)+\Gamma_{2}\left(\left\|s\right\|\right)s(t){\left\|s(t)\right\|}^{\beta -1}/\left({\left\|s(t)\right\|}^{1+\beta }+\mu \right)$$

*where 0<β<1, μ is a small positive constant, and nonlinear filtering gains $\Gamma_{1}\left(\left\|s\right\|\right)$ and $\Gamma_{2}\left(\left\|s\right\|\right)$ are designed as follows:*

(18) $$\Gamma_{1}\left(\left\|s\right\|\right)=\left[\bar{\alpha }/2+{\bar{\rho }}_{2}+(\rho_{1}+\sigma_{M}\zeta ){\left\|s(t)\right\|}^{\beta -1}\right]L_{1}>0$$

(19) $$\Gamma_{2}\left(\left\|s\right\|\right)=\left[\mu ({\bar{\rho }}_{1}+\sigma_{M}\zeta )+2^{-\beta }\bar{\beta }{\left\|s(t)\right\|}^{2\beta }\right]L_{2}>0$$

*where $L_{i}\in {ℜ}^{4\times 4}\geq I_{4},$ i=1,2, $\sigma_{M}=\underset{1\leq j\leq 4}{\max}\left\{\sigma_{j}\right\},$ and $\bar{\alpha }\geq \alpha^{\prime }\geq 0,$ $\bar{\beta }>0,$ where $\alpha^{\prime }\geq c_{2}\left\|\dot{\eta }(t)\right\|$.*

*The lumped uncertainties brought about mainly by the uncertain torque, saturated input, and the simplified dynamics (3) are expressed as follows:*

(20) $$\begin{array}{ll} \Pi (\eta ,\dot{\eta },\ddot{\eta },s,\tau ,t)= & -\tau_{u}(\eta ,\dot{\eta },\ddot{\eta },t)-\tau (t)+sat(\tau )\\ & -X_{nss}(\eta ,\dot{\eta })/2-(\chi -1)F(q)s(t) \end{array}$$

*It is assumed that the upper bound of the lumped uncertainties satisfies the following inequality:*

(21) $$\left\|\Pi (\eta ,\dot{\eta },\ddot{\eta },s,\tau ,t)\right\|\leq {\bar{\rho }}_{1}{\left\|s(t)\right\|}^{\beta }+{\bar{\rho }}_{2}\left\|s(t)\right\|\;$$

*where ${\bar{\rho }}_{1}$ and ${\bar{\rho }}_{2}$ are unknown positive constants.*

### 3.2. Stability Analysis

The properties of the nominal simplified AUV system (4), (5b), and (6) controlled by the proposed ARM-FTC (17)–(19) are addressed by the following theorem.

**Theorem** **1.** *Consider the system structure of a transformed nominal simplified AUV system (4), (5b), and (6) with the lumped uncertainties (20) satisfying the condition (21). Applying the proposed ARM-FTC (17)–(19) with adaptive law (11)–(14) to the above controlled system, the operating point converges to the zero auxiliary tracking error in fixed time, i.e.,*

(22) $$D_{s}=\left\{s(t)\in {ℜ}^{4}|\left\|s(t_{1}\leq t)\right\|=0\right\}$$

*where*

(23) $$t_{1}\geq t_{0}+\ln\left[1+\tilde{\alpha }{\bar{V}}_{s}^{1-\tilde{\gamma }}\left(t-t_{0}\right)/\tilde{\beta }\right]/\left[\tilde{\alpha }(1-\tilde{\gamma })\right]$$

(24) $$\tilde{\alpha }=\left({\bar{\alpha }}^{\prime }-\alpha^{\prime }\right)/\lambda_{m}\;$$

(25) $${\bar{V}}_{s}(t)=s^{T}(t)s(t)/2\;$$

(26) $$\tilde{\beta }={\bar{\beta }}^{\prime }/\lambda_{m}\;$$

(27) $$0<\tilde{\gamma }=\beta <1\;$$

*Here, $\lambda_{m}=\min\lambda \left\{\overset{⌢}{M}\right\}$ represents the minimum eigenvalue of $\overset{⌢}{M}.$ Moreover, $\left\{s(t),\eta (t),\tau (t),\hat{\phi }(t)\right\}$ are uniformly ultimately bounded (UUB) and the fixed-time tracking performance $D_{\eta }=\left\{e_{\eta }(t)\in {ℜ}^{4}|\left\|e_{\eta }(t_{2}\leq t)\right\|=0\right\}$ is achieved. Here,*

(28) $$t_{2}\geq t_{1}+\ln\left\{1+\alpha_{e}V_{e}^{1-\gamma_{e}}(t-t_{0})/\beta_{e}\right\}/\left[\alpha_{e}(1-\gamma_{e})\right]$$

(29) $$\alpha_{e}=2\lambda_{\min}\left(K_{1}+K_{2}/\upsilon \right)$$

(30) $$\beta_{e}=2^{\left(\alpha +1\right)/2}\left\|f_{n}\right\|$$

(31) $${\bar{\gamma }}_{e}=(\alpha +1)/2$$

**Proof.**  For simplicity, the arguments of variables are omitted. At the outset, the condition ${\bar{\phi }}_{m}\leq \hat{\phi }\leq {\bar{\phi }}_{M}$ is addressed. Consider the following Lyapunov function:

(32) $$V=V_{\tilde{\phi }}+V_{s}\;$$

where $V_{\tilde{\phi }}={\tilde{\phi }}^{T}\Psi^{-1}\tilde{\phi }/2$ and $V_{s}=s^{T}\overset{⌢}{M}s/2$. Taking the time derivative of (32) and using (9)–(21) yields

(33) $$\begin{array}{l} \;\dot{V}={\dot{V}}_{\tilde{\phi }}+{\dot{V}}_{s}\\ =-{\tilde{\phi }}^{T}\Psi^{-1}\dot{\hat{\phi }}+s^{T}\overset{⌢}{M}\dot{s}+s^{T}\dot{\overset{⌢}{M}}s/2\\ =-{\tilde{\phi }}^{T}\Psi^{-1}\left[\Psi (Z^{T}s-\hat{\phi }\sigma^{T}s{\left\|s\right\|}^{\beta })\right]+s^{T}\overset{⌢}{M}\dot{s}+s^{T}(\dot{\overset{⌢}{M}}-2\chi F)s/2+s^{T}\chi Fs\\ =-{\left(\bar{\phi }-\hat{\phi }\right)}^{T}(Z^{T}s-\hat{\phi }\sigma^{T}s{\left\|s\right\|}^{\beta })+s^{T}\overset{⌢}{M}\dot{s}+s^{T}(X_{nss}/2+\chi F)s\\ =-s^{T}\left[Z(\bar{\phi }-\hat{\phi })\right]+{\tilde{\phi }}^{T}\hat{\phi }\sigma^{T}s{\left\|s\right\|}^{\beta }+s^{T}\overset{⌢}{M}\dot{s}+s^{T}(X_{nss}/2+\chi F)s\\ =-s^{T}\left[\overset{⌢}{M}\left(\ddot{\eta }+\dot{s}\right)+F\left(\dot{\eta }+s\right)+\overset{⌢}{g}\right]+s^{T}Z\hat{\phi }+{\tilde{\phi }}^{T}\hat{\phi }\sigma^{T}s{\left\|s\right\|}^{\beta }+s^{T}\overset{⌢}{M}\dot{s}+s^{T}(X_{nss}/2+\chi F)s\\ =-s^{T}\left[\overset{⌢}{M}\ddot{\eta }+F\dot{\eta }+\overset{⌢}{g}\right]+s^{T}Z\hat{\phi }+{\tilde{\phi }}^{T}\hat{\phi }\sigma^{T}s{\left\|s\right\|}^{\beta }+s^{T}(X_{nss}/2+(\chi -1)F)s\\ =-s^{T}\left[\tau -\tau_{u}-(\tau -sat(\overset{⌢}{\tau }))-(X_{nss}/2+(\chi -1)F)s\right]+s^{T}Z\hat{\phi }+{\tilde{\phi }}^{T}\hat{\phi }\sigma^{T}s{\left\|s\right\|}^{\beta }\\ =-s^{T}\left[\Pi +\Gamma_{1}s+\Gamma_{2}s{\left\|s\right\|}^{\beta -1}/\left({\left\|s\right\|}^{1+\beta }+\mu \right)\right]+{\tilde{\phi }}^{T}\hat{\phi }\sigma^{T}s{\left\|s\right\|}^{\beta }\\ =s^{T}\left[-\Pi -\Gamma_{1}s-\Gamma_{2}s{\left\|s\right\|}^{\beta -1}/\left({\left\|s\right\|}^{1+\beta }+\mu \right)\right]+{\tilde{\phi }}^{T}\hat{\phi }\sigma^{T}s{\left\|s\right\|}^{\beta }\\ \leq \left[{\bar{\rho }}_{1}{\left\|s\right\|}^{1+\beta }+{\bar{\rho }}_{2}{\left\|s\right\|}^{2}-\left\|\Gamma_{1}\right\|{\left\|s\right\|}^{2}-\left\|\Gamma_{2}\right\|{\left\|s\right\|}^{{}^{1+\beta }}/\left({\left\|s\right\|}^{1+\beta }+\mu \right)\right]+\sigma_{M}\zeta {\left\|s\right\|}^{1+\beta }\\ =\left[\begin{array}{l} \left(-\left\|\Gamma_{1}\right\|+{\bar{\rho }}_{2}\right){\left\|s\right\|}^{1-\beta }\left({\left\|s\right\|}^{1+\beta }+\mu \right)\\ -\left\|\Gamma_{2}\right\|+\left({\bar{\rho }}_{1}+\sigma_{M}\zeta \right)\left({\left\|s\right\|}^{1+\beta }+\mu \right) \end{array}\right]{\left\|s\right\|}^{1+\beta }/\left({\left\|s\right\|}^{1+\beta }+\mu \right)\\ =-\left[\begin{array}{l} \left(\left\|\Gamma_{1}\right\|-{\bar{\rho }}_{2}\right){\left\|s\right\|}^{1-\beta }\left({\left\|s\right\|}^{1+\beta }+\mu \right)\\ +\left\|\Gamma_{2}\right\|-\left({\bar{\rho }}_{1}+\sigma_{M}\zeta \right)\left({\left\|s\right\|}^{1+\beta }+\mu \right) \end{array}\right]{\left\|s\right\|}^{1+\beta }/\left({\left\|s\right\|}^{1+\beta }+\mu \right)\\ =-\Sigma \left(\left\|s\right\|\right)\left\{\begin{array}{l} \left[\left(\left\|\Gamma_{1}\right\|-{\bar{\rho }}_{2}\right)-\left({\bar{\rho }}_{1}+\sigma_{M}\zeta \right){\left\|s\right\|}^{\beta -1}\right]{\left\|s\right\|}^{2}\\ +{\left\|s\right\|}^{-2\beta }\left[\left\|\Gamma_{2}\right\|-\mu \left({\bar{\rho }}_{1}+\sigma_{M}\zeta \right)\right]{\left\|s\right\|}^{2\beta }\\ +\mu \left(\left\|\Gamma_{1}\right\|-{\bar{\rho }}_{2}\right){\left\|s\right\|}^{1-\beta } \end{array}\right\}\;\\ \leq -\Sigma \left(\left\|s\right\|\right)\left\{\begin{array}{l} \left[\left(\left\|\Gamma_{1}\right\|-{\bar{\rho }}_{2}\right)-\left({\bar{\rho }}_{1}+\sigma_{M}\zeta \right){\left\|s\right\|}^{\beta -1}\right]{\left\|s\right\|}^{2}\\ +{\left\|s\right\|}^{-2\beta }\left[\left\|\Gamma_{2}\right\|-\mu \left({\bar{\rho }}_{1}+\sigma_{M}\zeta \right)\right]{\left\|s\right\|}^{2\beta } \end{array}\right\} \end{array}$$

where $0\leq \Sigma \left(\left\|s\right\|\right)={\left\|s\right\|}^{1+\beta }/\left({\left\|s\right\|}^{1+\beta }+\mu \right)<1.$ Based on the nonlinear filtering gains (18) and (19), the following inequality (34) can be obtained from (33) as

(34) $$\dot{V}+\Sigma \left(\bar{\alpha }{\bar{V}}_{s}+\bar{\beta }{\bar{V}}_{s}^{\beta }\right)=\dot{V}+{\bar{\alpha }}^{\prime }{\bar{V}}_{s}+{\bar{\beta }}^{\prime }{\bar{V}}_{s}^{\beta }\leq 0$$

where ${\bar{V}}_{s}=s^{T}s/2,$ $\Sigma \bar{\alpha }={\bar{\alpha }}^{\prime },$ and $\Sigma \bar{\beta }={\bar{\beta }}^{\prime }.$ From (34), $\left\{s,\eta ,\tau ,\hat{\phi }\right\}$ are UUB. Since $\left\{s,\hat{\phi }\right\}$ converge and are bounded, based on Lyapunov stability the following result is achieved:

(35) $${\dot{V}}_{s},\;{\dot{V}}_{\tilde{\phi }}\leq 0,\;\mathrm{as}\;0\leq t_{1}\leq t$$

where $t_{1}$ denotes the fixed time reaching to the zero auxiliary tracking error. Moreover, according to property P5, the following inequality can be obtained:

(36) $$\begin{array}{ll} \dot{V} & \leq {\dot{V}}_{s}=s^{T}\overset{⌢}{M}\dot{s}+s^{T}\dot{\overset{⌢}{M}}s/2\\ & \leq \lambda_{m}{\dot{\bar{V}}}_{s}+s^{T}(X_{nss}/2+\chi F)s\\ & \leq \lambda_{m}{\dot{\bar{V}}}_{s}+\alpha^{\prime }{\bar{V}}_{s} \end{array}$$

where $c_{2}\left\|\dot{\eta }\right\|\leq \alpha^{\prime }$ is a bounded and positive constant since $\left\{s,\eta ,\tau ,\hat{\phi }\right\}$ are UUB. From (34)~(36), the following inequality is achieved:

(37) $$\dot{V}+{\bar{\alpha }}^{\prime }{\bar{V}}_{s}+{\bar{\beta }}^{\prime }{\bar{V}}_{s}^{\beta }\leq \lambda_{m}{\dot{\bar{V}}}_{s}+\left({\bar{\alpha }}^{\prime }-\alpha^{\prime }\right){\bar{V}}_{s}+{\bar{\beta }}^{\prime }{\bar{V}}_{s}^{\beta }\leq 0$$

Alternatively,

(38) $${\dot{\bar{V}}}_{s}+\tilde{\alpha }{\bar{V}}_{s}+\tilde{\beta }{\bar{V}}_{s}^{\bar{\gamma }}\leq 0$$

where $\tilde{\alpha }$ and $\tilde{\beta }$ are described in (24) and (26). Based on Lemma 1, the fixed-time zero auxiliary tracking error, i.e., $s(t\geq t_{1})=0$, is accomplished. Then, from (9)

(39) $${\dot{e}}_{\eta }+K_{1}e_{\eta }+K_{2}\int_{t_{0}}^{t}e_{\eta }(\tau )d\tau +f_{n}sign{\left(e_{\eta }\right)}^{\alpha }=0$$

From (39), the following equation is obtained

(40) $${\dot{e}}_{\eta }=-\left[\left(K_{1}+K_{2}/\upsilon \right)e_{\eta }+f_{n}sign{\left(e_{\eta }\right)}^{\alpha }\right]$$

where υ≡d(⋅)/dt. Let us define the Lyapunov function $V_{e}=e_{\eta }^{T}e_{\eta }/2.$ Taking its time derivative and substituting (40) into it as well as using this property $sign{\left(e_{\eta }\right)}^{\alpha }={\left\|e_{\eta }\right\|}^{\alpha }sign(e_{\eta })$, it becomes

(41) $$\begin{array}{ll} {\dot{V}}_{e} & =-e_{\eta }^{T}\left[\left(K_{1}+K_{2}/\upsilon \right)e_{\eta }+f_{n}sign{\left(e_{\eta }\right)}^{\alpha }\right]\\ & \leq -2\lambda_{\min}\left[\left(K_{1}+K_{2}/\upsilon \right)\right]{\left\|e_{\eta }\right\|}^{2}\\ & -2^{\left(\alpha +1\right)/2}\left\|f_{n}\right\|{\left\|e_{\eta }\right\|}^{\alpha +1}/2^{\left(\alpha +1\right)/2}\\ & =-\alpha_{e}V_{e}-\beta_{e}V_{e}^{{\bar{\gamma }}_{e}} \end{array}$$

where $\alpha_{e},$ $\beta_{e},$ and ${\bar{\gamma }}_{e}$ are given in (29)–(31). Based on Lemma 1, the fixed-time zero tracking error is obtained. Modify (33) as follows:

(42) $$\begin{array}{ll} \dot{V} & \leq (33)-\delta {\left(\hat{\phi }-\bar{\phi }\right)}^{T}{\left\|s\right\|}^{1+\beta }\left(\hat{\phi }-{\bar{\phi }}_{M}\right)/\left(\mu +\left\|\hat{\phi }-{\bar{\phi }}_{M}\right\|\right)\\ & \leq (33)-\delta {\left(\hat{\phi }-{\bar{\phi }}_{M}\right)}^{T}{\left\|s\right\|}^{1+\beta }\left(\hat{\phi }-{\bar{\phi }}_{M}\right)/\left(\mu +\left\|\hat{\phi }-{\bar{\phi }}_{M}\right\|\right)\\ & \leq (33). \end{array}$$

It reveals that the right-hand side of (42) is more negative as compared with inequality (33) such that the boundedness of the learning parameters is assured. Likewise, the same result in (42) is obtained for the condition ${\bar{\phi }}_{m}>\hat{\phi }$. Therefore, two projections into the desired range of the learning vector ${\bar{\phi }}_{m}\leq \hat{\phi }\leq {\bar{\phi }}_{M}$ are accomplished. □

## 4. Simulations and Discussion

In the simulation, the notations and the physical parameter values of the AUV system are shown in Table 1. The initial position and orientation of the AUV are taken as ${\left[0-1\;0\;0\right]}^{T}.$ The desired trajectory $\eta_{d}(t)$ is assigned as ${\left[2\sin(0.5t)-2\cos(0.5t)0.1t0.5t\right]}^{T}$ and the external current disturbance ${\overset{⌢}{\tau }}_{u}$ is selected as ${\left[20\cos(100tx_{2})\;20\sin(200t)\;10\sin(40tx_{1})\;20\cos(0.1x_{4})\right]}^{T},$ which is huge and highly nonlinear. Part of it is brought up by the simplified transformed model (6). In addition, the initial value of the learning parameter is $\hat{\phi }(0)=0.01I_{32\times 1},$ where $I_{32\times 1}$ is a unit vector of dimension 32; ${\bar{\phi }}_{M}$ and ${\bar{\phi }}_{m}$ are, respectively, assigned as $30I_{32\times 1}$ and $-10I_{32\times 1}.$ The control parameters are chosen as α=0.85, β=0.85, $\varepsilon_{1}=0.1,$ $K_{1}=8I_{4},$ $K_{2}=6I_{4},$ $f_{n}=0.1I_{4},$ $\Psi =0.005I_{32},$ $\sigma =0.005I_{4\times 1},$ $\Gamma_{1}=\left(60+0.3{\left\|s(t)\right\|}^{-0.05}\right)I_{4},$ and $\Gamma_{2}=\left(5+0.1{\left\|s(t)\right\|}^{1.9}\right)I_{4},$ where $I_{n}$ is the unit matrix of n×n. From (10), the following components of the regression matrix $Z(\eta ,\dot{\eta },\ddot{\eta })=[z_{ij}],\;i=1,2,3,4,j=1,2,\ldots ,32\in {ℜ}^{4\times 32}$ are not unique and chosen as follows:

(43) $$\begin{array}{l} z_{1,1}=\left[{\ddot{\eta }}_{1}+{\dot{s}}_{1}\right]\cos^{2}x_{4};\;z_{1,2}=\left[{\ddot{\eta }}_{1}+{\dot{s}}_{1}\right]\sin^{2}x_{4};\;z_{1,3}=\left[{\ddot{\eta }}_{2}+{\dot{s}}_{2}\right]\cos x_{4}\sin x_{4};\;\\ z_{1,4}=\left[{\dot{\eta }}_{1}+s_{1}\right]x_{6}\cos x_{4}\sin x_{4};\;z_{1,5}=\left[{\dot{\eta }}_{1}+s_{1}\right]x_{8}\cos x_{4}\sin x_{4};\;z_{1,6}=\left[{\dot{\eta }}_{1}+s_{1}\right]\cos^{2}x_{4};\;\\ z_{1,7}=\left[{\dot{\eta }}_{1}+s_{1}\right]\sin^{2}x_{4};\;z_{1,8}=\left[{\dot{\eta }}_{2}+s_{2}\right]x_{8}\cos^{2}x_{4};\;z_{1,9}=\left[{\dot{\eta }}_{2}+s_{2}\right]x_{8}\sin^{2}x_{4};\;\\ z_{1,10}=\left[{\dot{\eta }}_{2}+s_{2}\right]\cos x_{4}\sin x_{4};\;z_{1,11}=x_{5}\left|x_{5}\right|\cos x_{4};\;z_{1,12}=x_{6}\left|x_{6}\right|\sin x_{4};\;z_{1,13}=0;\;z_{1,14}=0;\;\\ z_{1,15}=0;\;z_{1,16}=0;\;z_{1,17}=0;\;z_{1,18}=0;\;z_{1,19}=0;\;z_{1,20}=0;\;z_{1,21}=0;\;z_{1,22}=0;\;z_{1,23}=0;\;\\ z_{1,24}=0;\;z_{1,25}=0;\;z_{1,26}=0;\;z_{1,27}=0;\;z_{1,28}=0;\;z_{1,29}=0;\;z_{1,30}=0;\;z_{1,31}=0;\;z_{1,32}=0;\;\\ z_{2,1}=0;\;z_{2,2}=0;\;z_{2,3}=0;\;z_{2,4}=0;\;z_{2,5}=0;\;z_{2,6}=0;\;z_{2,7}=0;\;z_{2,8}=0;\;z_{2,9}=0;\;\\ z_{2,10}=0;\;z_{2,11}=0;\;z_{2,12}=0;\;z_{2,13}=\left[{\ddot{\eta }}_{1}+{\dot{s}}_{1}\right]\cos x_{4}\sin x_{4};\;z_{2,14}=\left[{\ddot{\eta }}_{2}+{\dot{s}}_{2}\right]\cos^{2}x_{4};\;\\ z_{2,15}=\left[{\ddot{\eta }}_{2}+{\dot{s}}_{2}\right]\sin^{2}x_{4};\;z_{2,16}=\left[{\dot{\eta }}_{1}+s_{1}\right]x_{8}\cos^{2}x_{4};\;z_{2,17}=\left[{\dot{\eta }}_{1}+s_{1}\right]x_{8}\sin^{2}x_{4};\;\\ z_{2,18}=\left[{\dot{\eta }}_{1}+s_{1}\right]\cos x_{4}\sin x_{4};\;z_{2,19}=\left[{\dot{\eta }}_{2}+s_{2}\right]x_{8}\cos x_{4}\sin x_{4};\;z_{2,20}=\left[{\dot{\eta }}_{2}+s_{2}\right]\cos^{2}x_{4};\;\\ z_{2,21}=\left[{\dot{\eta }}_{2}+s_{2}\right]\sin^{2}x_{4};\;z_{2,22}=x_{5}\left|x_{5}\right|\sin x_{4};\;z_{2,23}=x_{6}\left|x_{6}\right|\cos x_{4};\;z_{2,24}=0;\;z_{2,25}=0;\;\\ z_{2,26}=0;\;z_{2,27}=0;\;z_{2,28}=0;\;z_{2,29}=0;\;z_{2,30}=0;\;z_{2,31}=0;\;z_{2,32}=0;\;z_{3,1}=0;\;z_{3,2}=0;\;\\ z_{3,3}=0;\;z_{3,4}=0;\;z_{3,5}=0;\;z_{3,6}=0;\;z_{3,7}=0;\;z_{3,8}=0;\;z_{3,9}=0;\;z_{3,10}=0;\;z_{3,11}=0;\;\\ z_{3,12}=0;\;z_{3,13}=0;\;z_{3,14}=0;\;z_{3,15}=0;\;z_{3,16}=0;\;z_{3,17}=0;\;z_{3,18}=0;\;z_{3,19}=0;\;z_{3,20}=0;\;\\ z_{3,21}=0;\;z_{3,22}=0;\;z_{3,23}=0;\;z_{3,24}=\left[{\ddot{\eta }}_{3}+{\dot{s}}_{3}\right];\;z_{3,25}=\left[{\dot{\eta }}_{3}+s_{3}\right];\;z_{3,26}=x_{7}\left|x_{5}\right|;\;z_{3,27}=0;\;\\ z_{3,28}=0;\;z_{3,29}=0;\;z_{3,30}=0;\;z_{3,31}=0;\;z_{3,32}=0;\;z_{4,1}=0;\;z_{4,2}=0;\;z_{4,3}=0;\;z_{4,4}=0;\;\\ z_{4,5}=0;\;z_{4,6}=0;\;z_{4,7}=0;\;z_{4,8}=0;\;z_{4,9}=0;\;z_{4,10}=0;\;z_{4,11}=0;\;z_{4,12}=0;\;z_{4,13}=0;\;\\ z_{4,14}=0;\;z_{4,15}=0;\;z_{4,16}=0;\;z_{4,17}=0;\;z_{4,18}=0;\;z_{4,19}=0;\;z_{4,20}=0;\;z_{4,21}=0;\;z_{4,22}=0;\;\\ z_{4,23}=0;\;z_{4,24}=0;\;z_{4,25}=0;\;z_{4,26}=0;\;z_{4,27}=\left[{\ddot{\eta }}_{4}+{\dot{s}}_{4}\right];\;z_{4,28}=\left[{\dot{\eta }}_{1}+s_{1}\right]x_{6}\cos x_{4};\;\\ z_{4,29}=\left[{\dot{\eta }}_{2}+s_{2}\right]x_{6}\sin x_{4};\;z_{4,30}=\left[{\dot{\eta }}_{4}+s_{4}\right]x_{5};\;z_{4,31}=\left[{\dot{\eta }}_{4}+s_{4}\right];\;z_{4,32}=x_{8}\left|x_{8}\right|. \end{array}$$

**Figure 3:**
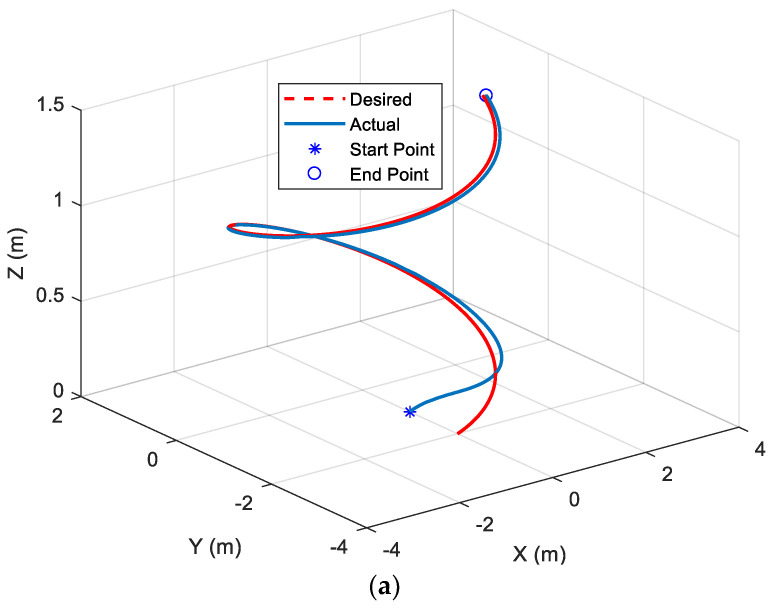

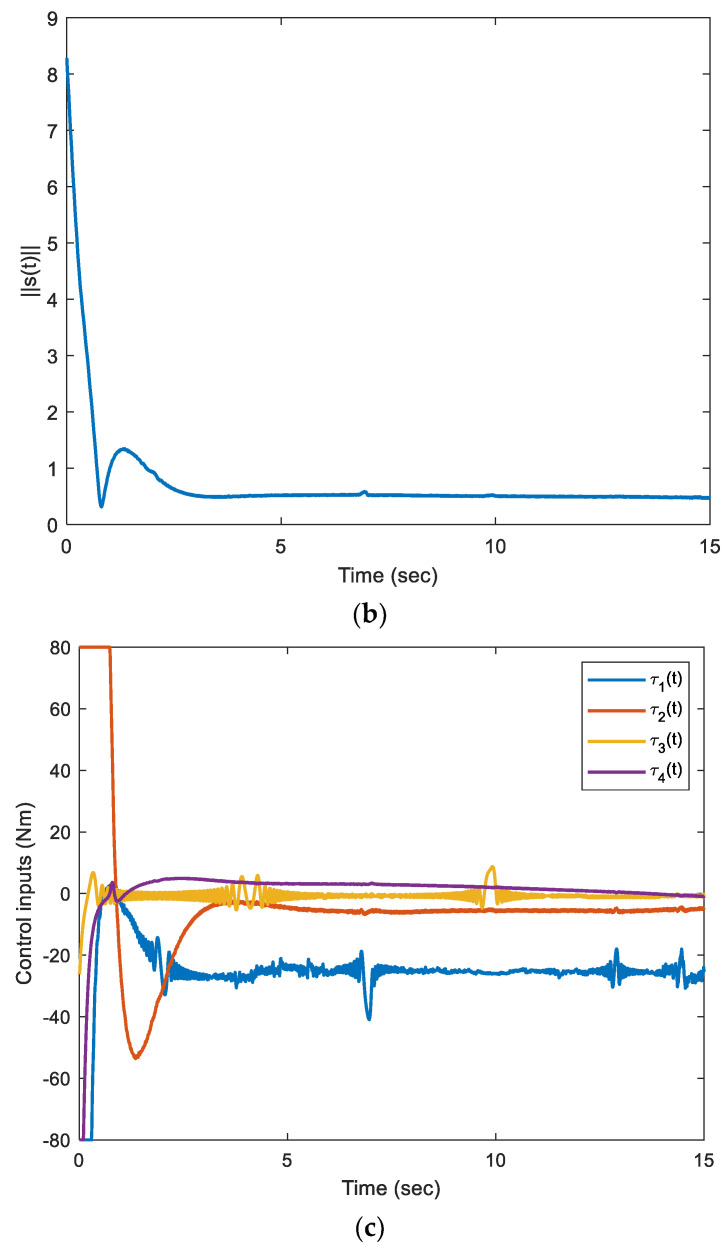
Responses of the AUV system in the presence of external current disturbance and input saturations with known parameters reflecting a regression matrix. (**a**) Trajectory−tracking. (**b**) Norm of nonlinear auxiliary sliding surfaces. (**c**) Control inputs.

The above non-unique regression matrix (43) is the key feature of this study, which is practical and effective. The response for the AUV system with the external current disturbance and input saturations and the known parameters reflecting a regression matrix are shown in Figure 3. Since the designed fixed-time control is applied to the AUV system, the 3D tracking performance in Figure 3a is satisfactorily achieved. Besides this, the norm of sliding surfaces shown in Figure 3b converges to the neighborhood of zero auxiliary sliding surfaces in fixed time. After the saturation at the initial time period, each component of the control inputs almost converges to a fixed value (see Figure 3c).

**Figure 4:**
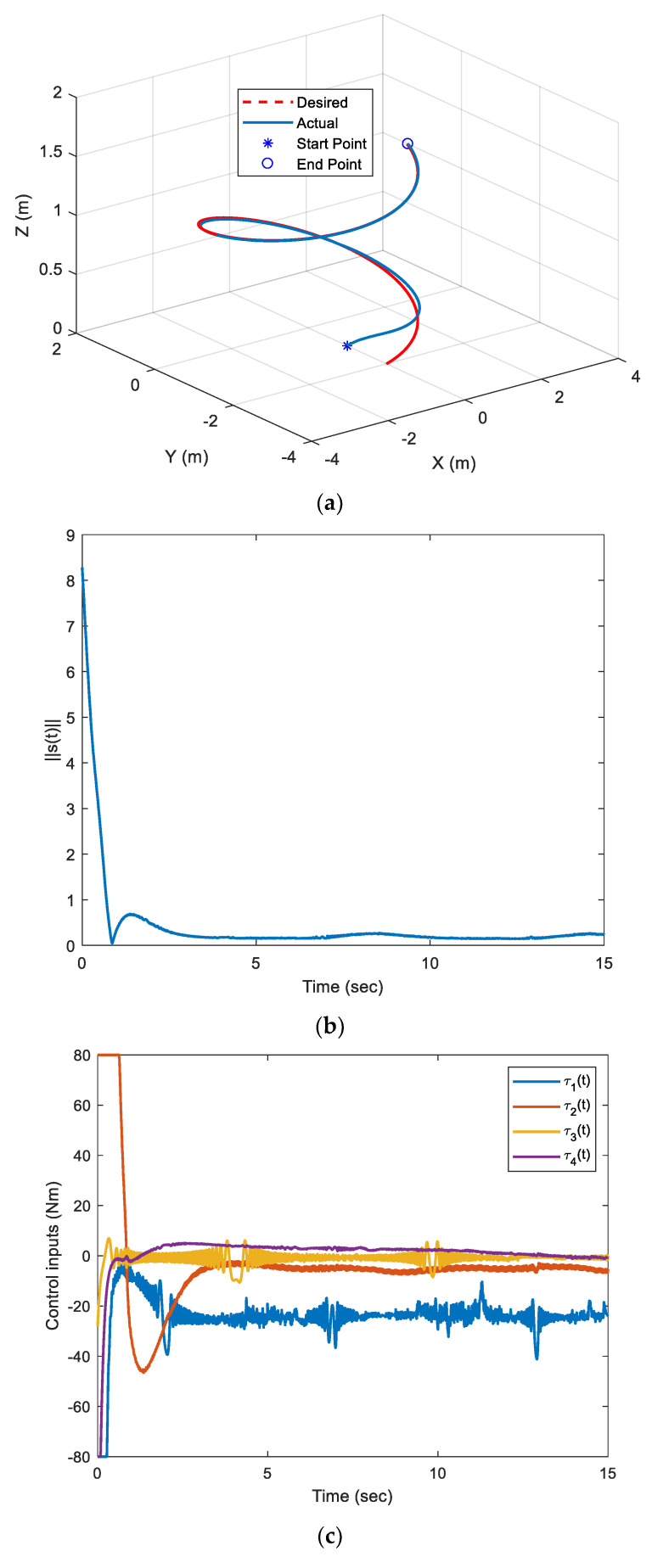

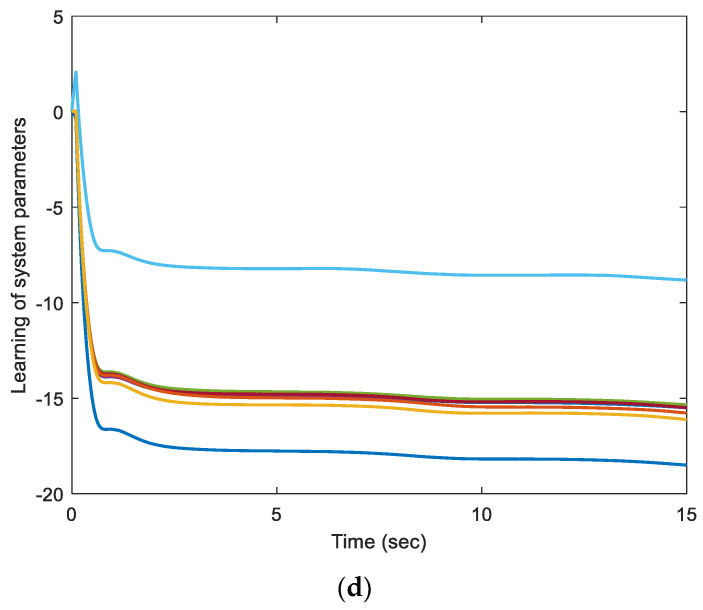
Responses of Figure 3 case with the proposed ARM-FTC. (**a**) Trajectory−tracking. (**b**) Norm of nonlinear auxiliary sliding surfaces. (**c**) Control inputs. (**d**) Learning parameters.

**Figure 5:**
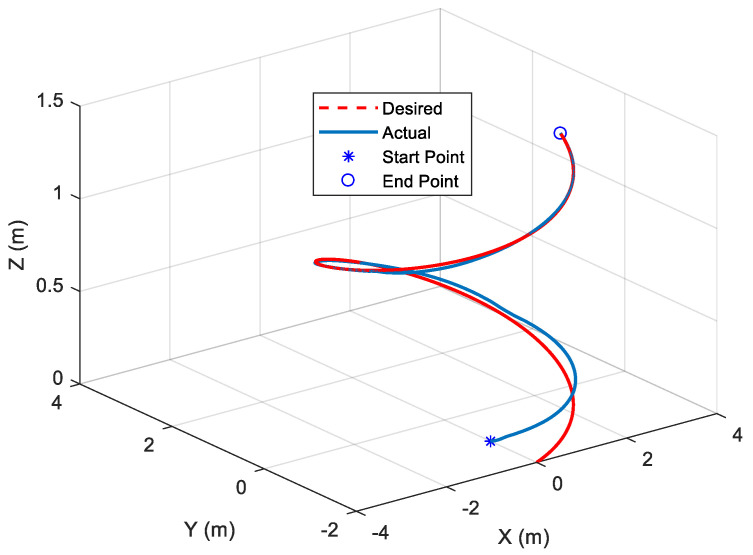
Tracking response of Figure 3 case with the robust asymptotical tracking control [26].

**Figure 6:**
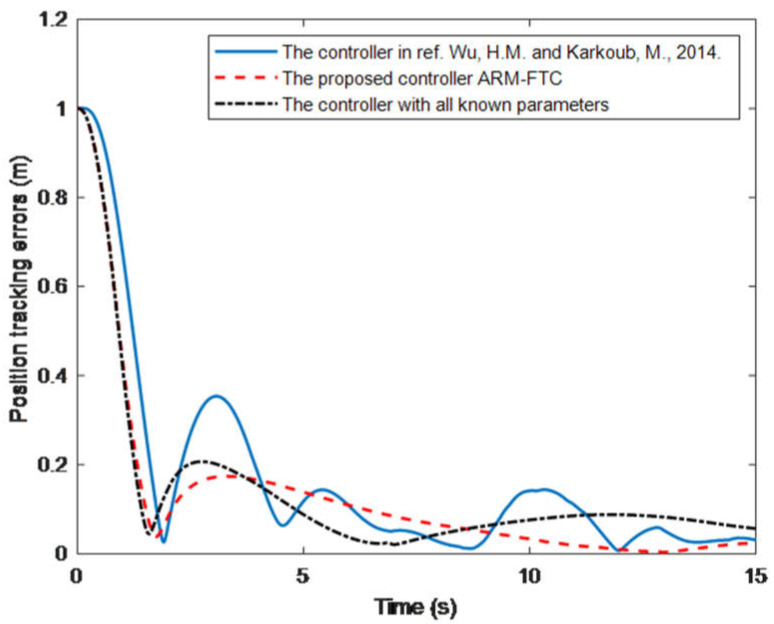
The comparisons of tracking errors for three methods [26].

Figure 4 shows the AUV performance when controlled by the proposed ARM-FTC. It can be seen that the proposed ARM-FTC can deal with the uncertainties including unmodeled dynamics to obtain a better tracking performance (cf. Figure 3a and Figure 4a or Figure 3b and Figure 4b). Figure 3b and Figure 4b not only converge to the neighborhood of the auxiliary sliding surface, but also the performance of Figure 4b is better than that of Figure 3b. In comparison to Figure 3c and Figure 4c, it is known that the proposed adaptive control can provide slightly high-frequency component of control inputs to improve the robust performance. That is to say, the proposed regression matrix-based adaptive control can deal with the unmodeled dynamics due to it resulting from the simplification of model (3). The behavior is also noticed in similar studies, e.g., the comparison of path tracking control of a car-like mobile robots with and without motor dynamics [25]. Figure 4d exhibits the learning parameters, which are bounded and converge to fixed values. Based on these responses, the proposed ARM-FTC not only effectively deals with a simplified AUV model in the presence of external current disturbances (including unmodeled dynamics) but also can mitigate the effects of input saturation. To further confirm the fast tracking ability of the proposed approach, the same system is controlled using the nonlinear trajectory-tracking control developed in [26], which is designed by the asymptotic tracking, and the results are shown in Figure 5. It can be clearly seen that the tracking response using the proposed ARM-FTC is superior to that using the previously developed control techniques for the simplified model of the AUV in the presence of input saturation and external current disturbances (cf. Figure 4a and Figure 5). Finally, the tracking errors of three methods are compared in Figure 6. Both transient and steady-state performances are better using the ARM-FTC technique. One major advantage of the proposed control technique is its applicability to a class of nonlinear control problems.

## 5. Conclusions

Through the appropriate transformation combining its kinematics with dynamics, a newly represented system model was achieved. This simplified model combined with the system structure was employed to describe the main characteristics of an AUV control system. The proposed adaptive regression matrix-based fixed-time controller (ARM-FTC) was designed here for the simplified AUV system model using a nonlinear auxiliary sliding surface. The surface was constructed using the tracking error to shape the system frequency response. It was shown that the proposed ARM-FTC can successfully achieve trajectory tracking by the AUV system despite the presence of uncertainties including unmodeled dynamics and input saturation. The uniform ultimate boundedness of the system states, control inputs, and learning parameters was demonstrated by the Lyapunov stability theory. In addition, fixed-time convergence of an auxiliary sliding surface and then zero tracking errors were achieved under an appropriate condition of lumped uncertainties. It was shown that compared to the results obtained using known parameters reflecting a regression matrix and robust asymptotical tracking control, the proposed ARM-FTC can tolerate unmodeled dynamics caused by simplification of the AUV system. In addition, implementation of the developed controller will be much easier compared to other similar techniques available in the literature. The future work is to implement the ARM-FTC on an AUV testbed.

## Figures and Tables

**Table 1:** Parameters of the AUV system.

	Property	Description	Value
Parameter			
m		Mass of the AUV	10 [kg]
B		Buoyancy force of the AUV	10 [NT]
$I_{z}$		Moment of inertia about the Z-axis of the AUV	$I_{z}=30\;\left[kgm^{2}\right]$
$x_{g},\;y_{g},\;z_{g}$		Position coordinate of the center of gravity	$x_{g}=0,\;y_{g}=0,\;z_{g}=6.1\;\left[m\right]$
$x_{b},\;y_{b},\;z_{b}$		Position coordinate of the buoyancy center	$x_{b}=0,\;$ $y_{b}=0,\;$ $z_{b}=0\;$ [m]
N		Component about the Z-axis of the total moment acting on the AUV (yawing moment)	
r		Angular velocity about the Z-axis (yawing)	
Added mass matrix			
$X_{\dot{u}}$		Partial derivative of X with respect to u	$X_{\dot{u}}=34$
$Y_{\dot{v}}$		Partial derivative of Y with respect to v	$Y_{\dot{v}}=75$
$Z_{\dot{w}}$		Partial derivative of Z with respect to w	$Z_{\dot{w}}=33$
$N_{\dot{r}}$		Partial derivative of N with respect to r	$N_{\dot{r}}=62$
Linear drag matrix			
$X_{u}$		Partial derivative of X with respect to u	$X_{u}=6$
$Y_{v}$		Partial derivative of Y with respect to v	$Y_{v}=10$
$Z_{w}$		Partial derivative of Z with respect to w	$Z_{w}=7$
$N_{r}$		Partial derivative of N with respect to r	$N_{r}=14$
Quadratic drag matrix			
$X_{uu}$		Components of the quadratic drag about the X -axis with respect to u	$X_{uu}=18$
$Y_{vv}$		Components of the quadratic drag about the Y -axis with respect to v	$Y_{vv}=4$
$Z_{ww}$		Components of the quadratic drag about the Z -axis with respect to w	$Z_{ww}=4$
$N_{rr}$		Components of the quadratic drag about the Z -axis of the total moment with respect to r	$N_{rr}=14$
